# Re-visioning intercultural relational empathy

**DOI:** 10.1007/s10459-025-10429-4

**Published:** 2025-06-04

**Authors:** Quentin Eichbaum, Alan Bleakley

**Affiliations:** 1 Department of Pathology, Microbiology and Immunology, Division of Medical Education and Administration, Vanderbilt University Medical Center, Vanderbilt University School of Medicine, TVC 4511C, 1301 Medical Center Drive, Nashville, TN 37232 USA; 2 Peninsula Medical School, Plymouth University, Plymouth, UK

**Keywords:** Empathy, Intercultural empathy, Relational empathy, Nomadic empathies, Empathic accuracy

## Abstract

We have previously argued that empathy is a multidimensional, context-modulated attribute, and that the Western unidimensional ‘one size fits all’ approach to empathy is inadequate particularly in intercultural settings. We called for relational empathy characterized by qualities such as curiosity; cultural and epistemic humility; bidirectional engagement; relational consciousness/ubuntu. In a paradigm shift from dominant models of ego-based empathy as projected content, here we describe a model of empathies or multiple ‘local stories’ that are process-based, fluid and context-dependent. Such empathies are not ‘given’ but ‘generated’ as an emergent property of social engagement based on a dialectic of democracy. Establishing such Intercultural Relational Empathies demands a shift from singular ‘content’ empathy to multiple ‘process’ empathies produced through sensitive, democratic encounter. We thus distinguish between ‘settled’ empathy as an individual trait and ‘nomadic’ empathies as negotiation in social settings. While the former describes empathy as ego-based content, the latter describes empathy as process and eco-centric – specifically, an emergent property of a nonlinear, open, dynamic, complex system that is an active social collective. We focus on the collective of an intercultural healthcare setting common to contemporary global healthcare - multicultural healthcare teams treating a range of patients. We describe ‘nomadic’ empathies as produced through dialectic and negotiation, both collaborative and competitive. We suggest that the globally dominant modernist model of content-based, ego-driven empathy is grounded in the values systems of individualism common to high-income countries. This affords a ‘grand narrative’ or dominant value, but one size does not fit all.

## Prologue: Two stories

Clinical practice benefits from framing encounters with patients in terms of narratives as well as technical data (Greenhalgh & Hurwitz, 1998). Here, then, are two stories. We write this at the height of the Israel-Hamas conflict in Gaza, with analogies to a contemporary Trojan War. Unsurprisingly, one clear phenomenon that this war has thrown up is how strongly partisan support is felt for one side or the other in the face of appalling atrocities on both sides. Empathy for many is experienced in one direction only - towards a favored side. For many onlookers globally, suspending identification with either side, there is surely a vast outpouring of unconditional and genuine empathy for the suffering and deaths of thousands of citizens. Which leads one to wonder, can empathy then be both unconditional (freely given) and conditional (given selectively)?

Kuper (2023: 14) reminds us that, after release from a long-term prison sentence, Nelson Mandela ‘modelled empathy across ethnic divides’, creating ‘a non-racial democracy’ – a ‘one-state solution’ where ‘South Africans of all colors accept each other as South African’. This advertises a ‘bi-directional’ empathy (3rd conversation 2022). Kuper calls supremely empathic persons such as Mandela ‘truth-seeking’ or authentic (and on ‘Team Humanity’). Here, ‘The rest are just cheerleaders’ with more timid or diluted empathy that we commonly call ‘sympathy’; or even displaying inauthentic empathy. Conditions are placed on empathy (or empathy becomes conditional). What is the key then to a deeper, unconditional ‘truth-seeking’ empathy? Can healthcare practitioners and clinical educators aspire to ‘Team Humanity’ or will we remain mere cheerleaders? Kuper suggests that the key to this deeper shift rests with unhooking oneself from partisan values, where empathy is but ‘a figment of your own identity’. Such ‘truth seeking in a time of tragedy’, as Kuper calls it, suggests again the Trojan War of Homer’s *Iliad*. Are you on one side or the other: Trojan or Greek, Hector or Achilles? Then your empathy will be partisan.

If you are neutral, do you show authentic empathy at all? The Trojans started the war by abducting Helen, while the Greeks finished it by smuggling in a small conquering army in the Wooden Horse. The parallels with the conflict in Gaza are contextually different and not clear-cut but nonetheless alluring – Hamas initiated conflict through committing atrocities and abducting hostages, while Israel reacted with overpowering aerial force. But to confront Hamas they will have to resort to Wooden Horse strategies of infiltration of tunnel systems. What species of empathies between partisans in the conflict are being constructed here will be unclear for some time, but empathies surely abound.

Let us now turn away from politics grounded in ethnicity and religion to the politics of medicine, and our second story. Everybody surely has empathy for those who need organ transplants and are on a waiting list for a suitable donor. Levels and authenticity of empathy may be affected by factors such as lifelong smokers waiting for a lung transplant or lifelong drinkers on the list for a liver transplant. Empathy suddenly becomes conditional.

Murgia (2023), the Artificial Intelligence (AI) editor of the *Financial Times*, shows how those waiting in the UK for a liver transplant are subject to an AI algorithm (obviously of human design) that has inbuilt bias against younger patients. In other words, even in the medical AI world, empathy is conditional, tempered by the harsh realities of a need to create a list of ‘most’ and ‘least’ urgent of all desperately urgent cases. As commodity, or capital, empathy-as-transplant (the ultimate gift of a donor and then the skill of the surgical team afforded to the recipient) is scarce and open to market forces.

But what kind of empathy are we describing? We assume that empathy has cognitive, affective, and compassionate expressions, but is ‘empathy’ necessarily personal capital (exercised from me as source to you as target)? We do not talk of multiple ‘empathies’ that are concurrently socially constructed and not owned by individuals and attached to ego (therefore usually making them conditional). Are there empathy constructions that are nomadic or fluid? Can an empathy be generated collaboratively (as process) that is qualitatively different from personal ego-empathy (as content)? Such empathy – viewed as shared capital - might be seen as a product of collaborative labour.

We see three intersecting dimensions to empathy as labour: the axiological (values), epistemological (conceptual), and ontological (experiential). In this article we consider all three dimensions as critical to the process work of collaborative making of ‘relational empathy’. Fagiano (2019) – who dislocates empathy from personal ownership into the social realm of ‘shared hope’ - claims to have introduced this descriptor, but it was first coined by Broome (1991), and later used by DeTurk (2001) as a ‘possibility for alliance building’. But surely all kinds of empathy are ‘relational’ by definition, as empathy is always shown to another or others? Yes, this directional tangent is true, but here we make a distinction that Broome, DeTurk and Fagiano do not make clear – that where empathy is shown by an individual towards another or others, it is personal capital issued from me to you, and often conditional. Such empathy content is not limitless, for empathy can decline (replaced for example by cynicism) or can run dry (‘I just have no feeling for her anymore’). Unhooking empathy from its ego or personal tether creates an axiological gap (whose values are we now discussing?), an epistemological query (how do we theorize empathies that are collectively produced and owned?), and an ontological conundrum (how are shared empathies experienced by individuals?) To further complexify empathy, we wish to add a key element: the intercultural. Conversations and translations both within subcultures and across cultures in the contemporary world of mass migration, ethnic conflict leading to displaced populations, global healthcare, fluid workforces, and mixed-ethnic patient populations. In a previous article we put flesh on the bones of an ‘intercultural relational empathy’ and pointed out through illustrative case studies some of the difficulties in exercising such empathy, and, importantly the dangers of exporting empathy styles from high-income to middle- and low-income countries as a form of imperialism or neo-colonialism (Eichbaum et al., 2023).

Here, we focus particularly on the axiological and epistemological dimensions to empathy by radically progressing our argument from the previous article (ibid.). We suggest that models of empathy (while acknowledging differences in style) are characteristically ego-based and source-to-target directed (where the source is the empathizer). We describe here a paradigm shift in understanding the dynamics of empathy by positing an ego-free empathy that is collectively produced and can be configured as process rather than content. This can be called a ‘moral empathy’ as a permeating value.

## From empathy as personal capital to empathy as shared capital

While ‘collective empathy’ is described as a shared phenomenon, it is typically also described from the individual’s perspective. Thus ‘emotional contagion’ describes affective identification with another’s feelings, while ‘perspective taking’ describes cognitive understanding of another’s perspective. Like a boomerang, empathy – even where described as ‘collective’ - returns as personal capital or a bolstering of identity (Lebow, 2019). An empathy shift from the personal to the relational demands unhooking from personal content and projected empathy. Here, also, empathy is not a quantity open to measurement, but a quality resisting measurement. It is an emergent product of collaborative inquiry, necessarily fluid and multifaceted in character. A deepening of such processual relational empathy occurs in cross-cultural exchanges, inviting translations. Here, all kinds of mistranslations, misunderstandings, and uncomfortable compromises are possible as well as successful negotiations.

The most common block to successful negotiation of shared intercultural empathy is the tendency to assume that empathy has a universally accepted meaning and is readily exported (Eichbaum et al., 2023). This modernist, ‘grand narrative’ (all-embracing theory) approach to empathy can be transformed into a postmodern, pluralist, and relativist approach composed of *petit récits* or ‘local stories’ (Lyotard, 1984). Not only do grand narratives shift to local stories, but personal empathy capital is reframed as shared, collective empathy capital. In summary, our suggested paradigm shift - from personal empathy to collectively constructed pluralistic empathies - reflects a shift from (i) modernism to postmodernism, (ii) imperialism to recovery of local identities, and (iii) capitalism (empathy as personal capital) to what Michael Hardt and Antonio Negri (2009) call ‘common wealth’ (empathy as shared capital).

In an intercultural setting at its core the making of relational empathy is not about acquiring a ‘skill’ or ‘competence’, but about negotiation of values positions - often of competing and intersecting values. A micro-example is the healthcare team, where the embracing ‘cultures’ in question are surgical, medical, and nursing; while a macro-example is Anne Fadiman’s (1997) *The Spirit Catches You and You Fall Down*, an account of the interactions between the conventional American healthcare system and a refugee family from Northwest Laos who had settled in California.

In the first example of the clinical team, axiological or values issues are the causes of tensions around collaborative production of empathy where, typically, nursing staff show high levels of empathy while doctors - surgeons in particular - show relatively low levels of empathy. These are two cultures who need to negotiate a shared empathy for patients and between themselves. Otherwise, in the personal empathy model and in a hierarchical system, the surgeons’ empathy style will typically dominate and possibly oppress (another form of imperialism) (Sier et al., 2022). How then will this multi-professional values conflict avoid the usual imperialist ‘solution’ of the imposition of hard-nosed values by the medical and surgical fraternity upon the nursing sorority in an undemocratic hierarchical team setting? How might democratic habits and values be gained to transform multi-professionalism into inter-professionalism?

One answer to this axiological dilemma – whose end-product can be paucity of empathy capital (exercised on behalf of the patient) for the team as a whole - is longitudinal work-based team education (Bleakley et al., 2006). This is critical for patient care, where paucity of collaborative empathy production is the basis for potential medical/ surgical error. There is a large literature demonstrating that an axiological shift towards democratic values in healthcare teams impacts positively not only on patient care and safety, but also on worker satisfaction (Edmondson, 2018).

In the second example – one of macro-intercultural relational empathy – the youngest daughter of the immigrant family is diagnosed with a severe form of epilepsy. A classic case of failure to create empathy capital through dialogue with the Hmong family on the part of the doctors treating the child leads to a case of axiological (values) imperialism. Mistrust and misunderstandings occur around patient adherence (or lack of it) such that the child’s condition worsens. The basic tension is between the family’s transcendental (meaning) values reading of the condition and the medical establishment’s instrumental-technical (material) reading. Spirituality and science fail to converse as potential empathy and subsequent insight bleed away.

The epistemological dimension to the production of intercultural relational empathy embraces theoretical models and abstractions that shape activities (the ontological or experiential dimension). Transition from a singular ‘one-size-fits-all’ values approach to empathy to a pluralistic model is demanded by collectivist and collaborative intercultural relational empathy. This is a dynamic process of production and does not treat various empathies as personal content (and capital) but as emergent properties of open, complex, adaptive systems that are collectives of healthcare professionals working around patients. Such production is often fraught with ambiguity and uncertainty. The ontological dimensions to empathy then follow, where empathy-as-given - as content and personal capital - transforms to empathy-in-construction, as process and ‘possibility knowledge’ (Engeström, 2018). Let us now track such a transition in more detail.

## Changing the climate to usher in a culture change (as paradigm shift)

A long-term collaborative inquiry research project - initiated by one of us (AB) at a hospital complex in the UK - investigated how surgical teams might improve teamwork for better patient care and safety, and to improve worker satisfaction (Bleakley et al., 2006, 2012, 2013). The central methodology was to videotape live teams at work and use key extracts from the video evidence in debriefing sessions. Researchers working with team members in debriefs also attended the surgical sessions in vivo and made field notes. Independently of these data collection methods, members of teams were interviewed using semi-structured methods. A serendipitous structuring within the hospital setting allowed for an experimental intervention with one set of surgical teams, while a similar number of teams in another part of the hospital afforded a control group. Here, the intervention was offered one year after initiation with the experimental cohort. Close call (or near miss) events (near accidents that could have resulted in injury or death to patients) were recorded across the two groups and analyzed for comparative content.

Every month, surgical team members in the experimental group attended a half day educational session where research data were presented and discussed, and strategies for change were developed. Every three months, theatre practitioners across both cohorts completed a validated Safety Attitudes Questionnaire that provided a snapshot of the safety climate across teams (such as levels of team relational empathy as a measure of interprofessional cohesion). Safety climate scores could then be compared. Team ‘climate’ shifts were seen as preliminary markers in a movement towards authentic democratising of team practices. This could be seen particularly in language exchanges where increasing frequency of dialogue rather than monologue showed a shift from hierarchical structures to democratic participation (seen also in increased quality of team briefing and debriefing).

The experimental team showed significant shifts over the control teams (that did not receive the educational intervention) towards establishing a democratic climate, marked by increased productive dialogue over monological exchanges. This was also shown in a significant difference between safety climate scores in the control and experimental groups, and between frequency and intensity of reported near misses (or close calls).

Video ethnographic observations, follow-up interviews, and de-briefing with the surgical teams showed that while individual empathy displayed by team members towards each other (read as a cross-cultural effect where nursing and medical cultures work together) was clearly important, much more important was the collective production of relational empathy as recognised by the team members in feedback and de-briefing sessions. This created an improved team climate and atmosphere, with a spin-off of improved work satisfaction. In other words, changes in values positively affected ingrained poor work habits. This would benefit patients directly, as the care and safety climate generated by the team process would cradle patients. Empathy generated by collaboration was tangibly different from personal empathy shown by individual team members, the latter either an inherent trait or practiced capability, the former a product of a process: situation-specific relations and exchange.

The contemporary clinical team is a microcosm of wider intercultural activity, for example where healthcare practitioners from high-income countries join healthcare teams in low-income countries; and where healthcare teams treat a wide ethnic mix of patients, including a high proportion of immigrants. In a previous article (Eichbaum et al., 2023), we gave examples of how ‘empathy dissonance’ can occur in both contexts. But again, this was in the context of describing an intercultural relational empathy that did not adequately shift the centre of gravity of empathy from content to process.

What leads to ‘dissonance/ disjunction’ in these intercultural encounters is mostly a lack of ‘empathic accuracy’. As such, hasty colonising acts are deferred - this gives a breathing space to negotiate the best possible care and safety for a patient within any given cultural context. This would define empathy within that context. Here, viewpoints about care now carry relative rather than definitive meanings. Advantages and disadvantages are open to negotiation within an overarching value system of tolerance for difference (underpinned by tolerance of ambiguity). In fact, here we will suspend use of the English term ‘difference’ (that often conjures up negative imperialist ghosts even where it may be used with good intentions). We prefer Jacques Derrida’s (1968) notion of *différance* - that fails to adequately translate into an English equivalent - that may be thought of as simultaneously recognising a difference and enacting deferral such that the difference is processed. In our example here, the deferral is the pause to suspend *content* acts of personal empathy (potentially intrusive in an intercultural setting) to shift to a more deliberate (and uncertain) *process* of production of empathy in collaborative inquiry. Such ‘process’ empathies are multifaceted and innovative.

As practitioners meet value-laden approaches, so they automatically suspend impulse and judgement to embrace the long tradition of cultural and moral relativism inherited from Friedrich Nietzsche, and the social empathy practices of the ophthalmologist, psychoanalyst and colleague of Sigmund Freud, Adler (2009). Adler, the father of community psychology and social work, coined the term ‘fellow feeling’ as a forerunner to ‘relational empathy’. This describes a shift from an ‘ego-logical’ to an ‘eco-logical’ (context-sensitive) perspective (Eichbaum et al., 2023). We thus democratise empathy and configure the process of empathy production as emotional labour within a wider social justice agenda.

As with all products of emotional labour, they are open to manipulation and deceit. ‘Empathy’ does not automatically signal authenticity. We have thus far painted a positive and optimistic – maybe idealist – picture of the potential to generate and share affect as collaborative empathy. This can be described as authenticity or congruence. But empathy can also be faked and abused. Indeed, Arlene Hochschild’s (1983) original use of the term ‘emotional labour’ referred not to collaborative production of affect (as in an a high-functioning clinical team, or a productive relationship between a patient and a healthcare provider for example), but to ‘faking it’ in adopting a social persona such as the ‘perfect housewife’. Hochschild describes emotional labour as: to ‘induce or suppress feeling in order to sustain the outward countenance that produces the proper state of mind in others’. This is what Erving Goffman (1956/1990) long ago called ‘impression management’– playing a social role that becomes an identity, where life is read as a drama (Goffman’s approach to social relations is called ‘dramaturgy’). This can extend to the common feelings that, for example, theatre nurses have historically managed in relation to microaggressions from (particularly) male surgeons, where the latter show little empathy, or even withhold empathy. These dynamics can be seen as forms of mistranslation across the divide of hierarchical models and democratic models (flattened hierarchies).

Let us give another example of mistranslation through an intercultural example and how this can frustrate the production of a collaborative empathy. A male Romanian patient in a UK hospital is about to undergo an angiogram requiring the insertion of a catheter in the groin. A translator is on hand to facilitate conversation between the cardiac intervention team and the patient. But there is no direct translation between the English word ‘groin’ and a Romanian equivalent. The doctor’s technical ‘inguinal’ area does not help. The closest translation is ‘between the legs’ – but this leads to understandable confusion (Bleakley, 2017). The young woman translator was unable to find a metaphor that bridged the communication gap, and the elderly male patient was understandably confused. Potential empathy collaboration was squashed by the emergent ‘mind the gap’.

We recognise the dangers of creating an opposition between ‘individualist’ and ‘collectivist’ approaches where such oppositionalism is a characteristic tactic of modernism, one of choosing sides (‘you are either with us or against us’). We do not wish to set up an oppositional category where moving beyond a modernist mindset is to abandon oppositionalism as a rhetorical tactic, embracing multiplicity and adherent contradictions. We recognise that individualistic and collaborative models may be held simultaneously. Our issue here is with the fact that the individualist model has become a dominant paradigm for high-income healthcare and is readily exported without adequate critical pause and reflection.

As ecological disaster, ethnic conflict, and forced political emigration (for reasons of persecution) become increasingly common in the global South, so there is increased immigration to high-income countries. In a healthcare context (with movement of patients to high-income countries and increasing supplement of healthcare provision in low-income countries by high-income countries’ healthcare staff) adaptation to such global flux requires abandoning a ‘one size fits all’ model of empathy inflicted through monologue, to a multiple model of empathies constructed through dialogue.

## Fostering intercultural relational empathy as engaging with an emergent social condition

A pedagogical model of personal instrumental ‘skill’ development (as content) is dominant in healthcare education – usually expressed as the acquisition of competences. But we see the shift to engaging with process-based intercultural relational empathy as an emergent property of a complex collaborative system. This again describes a paradigm shift in values rather than the acquisition of skill. In turn, this brings pedagogical challenges such as, can we ‘teach’ the production of intercultural relational empathy that we model here as an emergent property of a complex system? The short answer to this is ‘no’. But what we can do is set up the conditions of possibility for engagement with the empathy paradigm shift that we describe as essential to an emerging healthcare climate. This is to *foster* such empathy. While we can certainly prepare practitioners to engage with the process of the social emergence of empathy, why we cannot simply ‘teach’ a set of skills or competences is illustrated by the case of Filipino nurses below.

In a global context of fluid healthcare workforces, Filipino nurses have become a key factor, often treated as a vital commodity (Montayre et al., 2021; Reyes et al., 2022). Such nurses provide the bulk of migrant labour in healthcare communities globally, yet they afford challenges in terms of integration into existing healthcare cultures. On the one hand, as Reyes and colleagues (ibid.) note: ‘like people in most Asian countries, Filipinos are collectivists in their cultural orientations; in contrast, most Western cultures, such as the UK, are individualistic’. This might suggest that Filipino nurses would welcome democratic teamwork, where Reyes and colleagues (ibid.) also note that such nurses value ‘community more than themselves as individuals’.

However, as the authors note, ‘people from collectivist cultures have tremendous respect for authority’. Filipino nurses prefer autocratic structures and shy away from public debate. Work satisfaction is provided by structure rather than risk and innovation. Further, censure in public is seen as humiliating, where such nurses ‘prefer feedback from a supervisor to be done in private’ (Reyes et al., 2022: 2). Typically, then, Filipino nurses – in an intercultural context - expect to fit into multi-professional hierarchies rather than inter-professional democracies, simultaneously subscribing to a collectivist spirit. They then remain perplexed at progressive working habits that develop authentic inter-professional democracies. Their collectivism is conservative rather than progressive.

The extent to which empathy can be taught remains a long-standing debate in Western medicine. For example, where Helen Riess (2022) emphatically claims that ‘Empathy can be taught and learned with evidence-based education’, in contrast Macnaughton (2009) claims that professional ‘empathy’ between doctors and patients is a flawed notion, a philosophical conundrum, and a pedagogical blind alley. Macnaughton argues that in a clinical setting a doctor can never truly stand in the shoes of the patient. Riess progresses her argument where she makes a distinction between values informing empathy and empathy as a professional stance: ‘Physicians can learn empathy skills that are perceived by patients without significant shifts in their attitudes’. The bottom line in nearly all studies of empathy is that the ‘empathy’ under consideration is what empathy tests, such as the Jefferson Scale, measure. How will such tests ‘measure’ multiple, fluid, and competing ‘empathies’ subject to context as ‘local stories’? Where Sulzer and colleagues (2016) conceptualise empathy as an engagement between subjects that is relational and comprised of dynamic interacting multi-dimensional components, we are of course empathic with such an approach! How then do we measure the *quality of an interaction* rather than *the quantity of an attribute*?

For this, we must shift perspective from object-already-found as content, to object-in-constant-creation as process. We need a processual or a ‘translational’ approach. There is a precursor in the work of Engeström (2008) on clinical teamwork, who uses neologisms describing activities as process rather than content, such as ‘teeming’ rather than teamwork, and ‘knotworking’ for temporary but productive encounters between members of teams. The linguistic device (translations of nouns into verbs) acts as a more authentic descriptor of what is happening on the ground but is also rhetorical in nature.

Yates-Doerr (2018) describes a ‘translational competency’, where:

A premise of translational competency is that, because interactions are dynamic, it is simply not possible to communicate without transforming meanings. To be competent in translation is to maintain awareness of the necessary equivocation that happens in interactions, where people speak using terms that will not mean the same thing between speakers, and which do not, in fact, have fixed meanings at all.

(Although we argue that ‘capability’ is a more appropriate descriptor than ‘competence’, particularly for a fluid notion). Indeed, Yates-Doerr puts her finger on the core issue that we discuss in this article. She sees two levels to addressing the issue. The first, as we suggest here, is to change paradigms of perception and understanding from a linear, problem solving, instrumentalist approach (that we have aligned with classic modernism and its major trope of oppositionalism) to a complex, problem-stating approach. The second level is to become proficient in translation-as-empathy across contexts. De Turk (2001), Sulzer and colleagues (2016), and Decety (2020) argue against empathy as an individually acquirable skill or competency, emphasising that it is context specific and relational. This is not just literally translating across languages and their semiotics, but also translating across subcultures within a culture – as we have noted, for example, translating between medicine and nursing subcultures. An issue that arises here is that, in negotations, how we will aid ‘empathic accuracy’ (establishing common targets)?

## Empathic accuracy

There are many factors that create dissonance in relational empathic encounters, such as inauthentic empathy (common in ‘professional’ communication), over-empathy (swamping with affect, treacly or sticky affect, or distancing through incisive perceptions – ‘you know me too well!’), and under-empathy as sympathy: ‘I know what you mean, but I don’t feel it’). These are largely conveyed through non-verbal cues such as eye contact, tone of voice, posture, proximity, and gesture (see later). These can be seen as failures in ‘empathic accuracy’ (Ickes et al., 1990) in both giving and receiving.

While empathy may be inauthentic (again, for example, ‘professional’ empathy administered when a healthcare professional is too busy or exhausted to properly attend to a patient) this is not a deliberate faking of empathy. Here, empathy is feigned (not necessarily with malintent). As noted above, the Scottish General Practitioner and medical educator Jane Macnaughton, a long-time champion of the medical humanities, makes the radical claim that all professional (as opposed to personal or familial) empathy is necessarily feigned, because nobody can truly put themselves in another person’s shoes. In a razing of years of hand wringing over empathy by medical and healthcare professionals, Macnaughton (2009) claims that empathy is a short-lived experience in clinical contexts and then in essence inauthentic, while empathy in intimate personal encounters is tangibly different, and patently authentic: ‘A doctor who responds to a patient’s distress with “I know how you feel” is likely, therefore, to be both resented by the patient and self-deceiving’. Kumagai (2008: 657) warns against teaching ‘empathy’ to medical students, suggesting that students may benefit more from learning a generalised ‘perspective taking’ as part of acquiring a wider narrative sensibility. Clinical thinking with the patient’s narrative in mind is of course quite different from imposing a medical narrative on the patient.

Different from lack of empathy is misconstrued empathy, empathic ‘dissonance’, and its more severe form ‘empathic disjunction’ (Eichbaum et al., 2023). When empathy goes wrong - is miscommunicated and/or misinterpreted - there is a lack of empathic accuracy. Loosely defined, empathic accuracy encompasses both the authenticity and the reception of empathy. ‘Accuracy’ here refers to how well the empathiser understands and identifies with the other. Zaki and colleagues (2008) enlarge Ickes and colleagues’ (1990) notion of ‘empathic accuracy’ to move from personal to ‘interpersonal’ empathy, as the product of an exchange. Empathy is not just given but felt and responded to. Empathic accuracy here depends on both the target and perceiver of empathy. In the personal model of empathy, levels of accuracy (and then inaccuracy) are dependent upon the quality of relationship between sender and receiver, the content of the exchange (for example is cognitive or affective empathy involved), and at what intensity (affective state) is the exchange experienced by each party? From a relational empathy point of view, such empathy ‘accuracy’ models appear strained. ‘Accuracy’ might be seen not as a trait of the empathiser, or as a perception of the empathiser by the Other, but as the quality of a process of co-relation or communal effort. ‘Accuracy’ is perhaps the wrong term to use when we are talking of moving targets in the emergence of processual, collective empathy.

‘One-size-fits-all’ individual empathy models might embrace interaction, but they fail to illuminate the dynamic process of empathy building, with its inevitable contradictions and complexities. Further, such contradictions can be seen as opportunities - for they are the grit that makes the pearl in the process of intercultural empathy. If empathies are context-sensitive, then the more divergent the empathic contexts are, the greater the potential capacity for empathic dissonance, disjunction, or inaccuracy. This is the key dilemma in cross-cultural empathic encounters where the gap between contexts is widest and thus more prone to misinterpretation and inaccuracy. Empathic accuracy could be enhanced if it was not just interactional, but co-directional and dynamically relational – in other words, preparatory groundwork is carried out for engaging with a dynamic social context without projecting self-interests.

Broome (1991) suggested that in a professional relationship (as between healthcare worker and patient) we go through ‘an infinite series of successive approximations’ as the co-construction of a shared world of understanding and meanings, as ‘relational attunement’. This likely enhances empathic accuracy and it surely follows the movements of every democratic dialectical conversation. Thus, we will have empathy theses, antitheses, and subsequent syntheses. Contradictions are inherent to such dialectic but can be taken as opportunities rather than threats (Engeström, 2018). Another way of looking at empathic accuracy is then ‘democratic attunement’.

Establishing such democratic attunement in relational empathy entails eco-logical (worldly and just) attributes such as curiosity, humility, a capacity for tentativeness, playfulness, and tolerance of uncertainty, rather than an ego-logical drive towards certainty, closure and control (Phillips et al., 2013; Eichbaum et al., 2023). For instance, curiosity - through its questioning and explorative qualities, and performed mutually and with humility - fills the gaps in understanding between individuals in reaching shared understanding. We have suggested that contradictions inherent to negotiations of a collaborative empathy offer an opportunity rather than a threat. They slow down over-hasty impulses to colonise another’s viewpoint.

## Skeletal empathy

To identify the key attributes/features of intercultural relational empathy, one might begin by asking what is missing when there is a lack of empathy or when empathy goes awry – when there is no flesh on the bones, so that empathy is at a bare minimum or skeletal. For example, ‘concern’ is not the same as empathic insight. Handwringing, or ‘tea and sympathy’ are certainly not the same as being able to stand in another’s shoes. We have already given examples of skeletal empathy: ‘empathy decline’ amongst later years medical students is like the flesh of deep human concern falling away to reveal the skeleton that is functional medicine, underpinned by competency approaches. ‘Good enough’ empathy may decline to cynicism.

Surgeons, amongst medical professionals, have been reported as lacking empathy (or not seeing empathy as particularly important to doing the job well) in comparison with other specialty doctors and healthcare professionals (Sier et al., 2022). For example, a study from Pakistan reports significantly lower scores by surgeons compared with other medical specialties as measured on the Jefferson Empathy Scale (Rashid et al., 2021). Also, a recent study of over 800 orthopaedic surgeons in Canada showed that, when rated on a scale of relational empathy, 50% of patients did score their surgeons highly, which is good news. But the bad news is that 50% of patients felt differently, giving only weak to moderate scores on relational empathy (Dobransky et al., 2020). Han and Pappas (2017) conducted a literature review of studies of empathy amongst surgeons, echoing a common refrain and suggesting a cure:

There is evidence of a decline in empathy that begins during the clinical years of medical school, which continues throughout residency training. Surgeons are particularly susceptible to this decline as by-product of the nature of their work, and the current lack of formalised training in empathic patient communication poses a unique problem to surgical residents.

There are many reasons for this historically ingrained symptom of empathy lack in surgery. Surgery is a technique-oriented craft culture occupied traditionally by men. Characteristically, medical students who do not orient themselves towards the communication and support - or care - side of medicine, but are more interested in a focus on cure, will follow certain specialty paths such as surgery, radiology, or pathology, as opposed to paediatrics, gerontology, or family medicine. Understandably, empathy decline is also a direct result of burnout, an increasingly common phenomenon amongst junior doctors as medicine becomes more complex, demanding, and stressful with pressures on resources (Peterkin & Bleakley, 2017). This is a chicken-and-egg problem: is empathy decline a result of, or the cause of, increase in stress? Whatever the reasons for lack of empathy or empathy decline, in both cases the pre-conditions for engaging with collaborative building of empathy are compromised.

## The empathy wars

We have repeatedly pointed out that ‘empathy’ is a troublesome notion, open to many interpretations, despite attempts to operationalise a complex of qualities in uniform, linear quantitative empathy scales. The focus here is on the modernist ideal of empathy as a personal quality or capital (product). Our significant move in this article is to re-define empathy as a postmodern process of negotiated, multiple, fluid, relative, competing ‘local narratives’ as opposed to an overriding grand narrative. Empathy can then be seen as an emergent property of such an open, dynamic, problem-stating, complex system, rather than a linear, closed, problem-solving device. Empathy may be seen as perplexing, nomadic and shape shifting rather than a territorial imperative disguised as doing good. As a multi-faceted process, relational empathy is a product of collaborative inquiry and democratic dialogue. Empathy is also now shared capital, accrued as ‘common wealth’ (Hardt & Negri, 2009) amongst stakeholders (such as clinical team members). This is quite different from empathy held as personal capital.

Through this revisionist lens, it is therefore frustrating for us to see ‘empathy wars’ at work in the literature that merely warms over what is already known, rather than breaking new ground, as ‘possibility knowledge’ and ‘spearheads of innovation’ to draw again on Yrjö Engeström’s (2008, 2018) future-facing vocabulary. For example, Jeremy Howick and colleagues (2023) have recently carried out a systematic review of the literature on the long-documented decline in empathy amongst medical students. The authors claim that their study ‘is the first to systematically demonstrate why empathy declines during medical training and raises important questions about the priorities of current medical education’. They suggest that:

Increased complexity in patients and their diseases, together with the ‘hidden curriculum’ (including a stressful workload, prioritisation of biomedical knowledge, and (sometimes) poor role models), led to student adaptations, such as cynicism and desensitisation. Students’ prior lives and professional experiences appeared to exacerbate the decline in empathy.

While these conclusions seem to us to re-state familiar arguments, staked out in familiar territory, our worry is not with the hyperbole of the claim for innovation, but with the fact that the reading of ‘empathy’ is modernist and unreconstructed. The problem rests with what Gilles Deleuze and Félix Guattari (1980/2013) refer to as the differences between ‘nomadic’ and ‘territorialising’ activities. The territorial mentality is to constantly revisit and refine the known through slight adjustments to borders and boundary markers. We think this is what Howick and colleagues’ study does. In contrast, de-territorialising approaches break free of constraining models to again pursue ‘spearheads of innovation’. We think that intentionally collaborative, process-related relational models do this. These ideas are nomadic/ unsettled rather than settled/ constrained. We fear that the empathy debate has lost its way, is running out of nourishment, and becoming skeletal. We hope that this article puts some flesh on the bone.

## Data Availability

No datasets were generated or analysed during the current study.
